# Pattern of inappropriate antibiotic use among hospitalized patients in Pakistan: a longitudinal surveillance and implications

**DOI:** 10.1186/s13756-019-0649-5

**Published:** 2019-11-21

**Authors:** Zikria Saleem, Hamid Saeed, Mohamed Azmi Hassali, Brian Godman, Usama Asif, Mahrukh Yousaf, Zakiuddin Ahmed, Humayun Riaz, Syed Atif Raza

**Affiliations:** 1 School of Pharmaceutical Sciences, Universiti Sains Malaysia, Gelugor, Malaysia; 2 Hamdard Institute of Pharmaceutical Sciences, Hamdard University, Islamabad, Pakistan; 3 University College of Pharmacy, University of the Punjab, Lahore, Pakistan; 4 Division of Clinical Pharmacology, Karolinska University Hospital Huddinge, Karolinska Institute, Stockholm, Sweden; 5 Strathclyde Institute of Pharmacy and Biomedical Sciences, Strathclyde University, Glasgow, UK; 6 Health Economics Centre, University of Liverpool Management School, Liverpool, UK; 7 Medical Centre, Agha Khan University Hospital, Karachi, Pakistan; 8 Ripha Institute of Healthcare Improvement & Safety, Ripha University, Islamabad, Pakistan; 9 Rashid Latif College of Pharmacy, Lahore, Pakistan

**Keywords:** Appropriateness, Antibiotics, hospitals, Prescribing, Pakistan

## Abstract

**Background:**

The inappropriate use of antibiotics in hospitals increases resistance, morbidity, and mortality. Little is currently known about appropriate antibiotic use among hospitals in Lahore, the capital city of Pakistan.

**Methods:**

Longitudinal surveillance was conducted over a period of 2 months among hospitals in Lahore, Pakistan. Antibiotic treatment was considered inappropriate on the basis of a wrong dosage regimen, wrong indication, or both based on the British National Formulary.

**Results:**

A total of 2022 antibiotics were given to 1185 patients. Out of the total prescribed, approximately two-thirds of the study population (70.3%) had at least one inappropriate antimicrobial. Overall, 27.2% of patients had respiratory tract infections, and out of these, 62.8% were considered as having inappropriate therapy. Cephalosporins were extensively prescribed among patients, and in many cases, this was inappropriate (67.2%). Penicillins were given to 283 patients, out of which 201 (71.0%) were prescribed for either the wrong indication or dosage or both. Significant variations were also observed regarding inappropriate prescribing for several antimicrobials including the carbapenems (70.9%), aminoglycosides (35.8%), fluoroquinolones (64.2%), macrolides (74.6%) and other antibacterials (73.1%).

**Conclusion:**

Educational interventions, institutional guidelines, and antimicrobial stewardship programs need to be developed to enhance future appropriate antimicrobial use in hospitals in Pakistan. Policies by healthcare and Government officials are also needed to minimize inappropriate antibiotic use.

## Background

The role of antibiotics in successfully treating infectious diseases cannot be denied. However, inappropriate antibiotic use poses a major challenge to public health in terms of increasing antimicrobial resistance (AMR) with its associated impact on morbidity, mortality, and costs as well as increasing adverse effects [1]. This has resulted in a number of activities globally, regionally, and nationally to reduce rising rates of AMR [2].

Whilst antibiotics are principally prescribed in ambulatory care [3], antibiotics are still frequently prescribed in hospitals. The Centers of Disease Control (CDC) in the US reported that 55.7% of patients from 323 hospital settings were given antibiotics during their hospital stay [4]. There is also high use of antibiotics among hospital patients in low and middle-income countries (LMICs), enhanced in some countries by high rates of HIV, TB, and malaria among in-patients [5–7]. However, there are concerns about the extent of inappropriate prescribing of antibiotics in hospitals [8]. According to a hospital survey in the US, 576 out of 1941 antibiotic therapy days were not essential due to the unnecessary length of prescribing, use of antibiotics for non-bacterial purposes and the treatment of colonizing agents [9]. In Romania, 49.2% prescriptions for antibiotics in an academic hospital were inappropriate on the basis of incorrect doses, duration and wrong indications [10]. Concerns with inappropriate prescribing of antibiotics in hospitals are enhanced by incentives to physicians, limited physician knowledge regarding antibiotics, and pharmaceutical company activities in some countries [11, 12].

In LMICs, physicians are particularly challenged with antimicrobial prescribing due to a lack of diagnostic facilities and poor level of healthcare systems in a number of countries as well as a lack of antimicrobial stewardship programs [13, 14]. As a result, high rates of antimicrobial resistance have been seen in LMICs [1, 2]. In a study among private hospitals in Lahore, Pakistan, the authors found that out of 93 *Escherichia coli* isolates, 82% were resistant to beta-lactam antibiotics with many resistant to fluoroquinolones and trimethoprim-sulfamethoxazole – attributable to antibiotic overuse [15]. However concerns with antimicrobial prescribing in LMICs are not universal with 62% of prescriptions for antibiotics in Namibia complying with national guidelines, although below the target of 95%, and ceftriaxone was appropriately prescribed in hospitals in Ghana [16, 17].

However, the frequent and needless use of antibiotics has increased AMR rates in Pakistan [18]. There have been published studies documenting high inappropriate use of antibiotics in ambulatory care in Pakistan [19]. There have also been a limited number of studies assessing antibiotic use in hospitals in Pakistan [6, 20]. However, we are not aware of any studies that have fully evaluated the appropriateness of antimicrobial prescribing among hospitals in the capital city of Pakistan to provide guidance to all key stakeholder groups. Consequently, we sought to address this.

## Methods

### Study design and settings

We conducted a longitudinal observational study over 2 months between March and April 2017 among two private hospitals and two public sector hospitals in Lahore, Pakistan, the capital city of Pakistan. This reflects the availability of both private and public sector hospitals in Pakistan. We believed that if we saw major issues regarding antimicrobial prescribing among hospitals in the capital city, Lahore, this would be reflected in hospitals throughout Pakistan. The participation of hospitals was voluntary. Antibiotics were prescribed in each hospital by the physicians in charge of the patients’ care, based on their own decision, or based on recommendations of the consultant, with currently no hospital or national guidelines to guide prescribing. The diagnosis was based on clinical judgement as typically culture and sensitivity testing (CST) is not undertaken in hospitals in Pakistan unless patients are in a critical condition in view of financial constraints. Patients undergoing treatment with one or more antibiotics either for a perceived clinical infection or surgical prophylaxis were included in the study.

We assessed the patients who were hospitalized in pulmonology, gastroenterology, nephrology, orthopedic, and general surgical/medical wards. We excluded those wards where antimicrobial prescribing patterns were seen as same in all patients, e.g., all patients were given ceftriaxone and metronidazole as prophylaxis in gynecological and obstetric wards. Patient receiving antimicrobials for medical prophylaxis were also not included because the indication was not clear. We also excluded all those wards where systemic antimicrobial use was perceived to be very low such as eye wards and Ear Nose and Throat (ENT) wards.

### Data collection

Patients were categorized according to International Classification of Diseases (ICD) as being treated for a site-specific infection such as a respiratory tract infection, including lower respiratory tract infections such as pneumonia, tuberculosis, and exacerbations of chronic obstructive pulmonary disease; a urinary tract infection including cystitis and pyelonephritis; a skin and soft tissue infection including bursitis, wound infection and cellulitis; or a bloodstream infection.

Experienced clinical research coordinators were trained to collect data prior to initiating the survey. Hospital pharmacists coordinated the data collection in each ward, while physicians and nurses also helped with data collection. Data were collected from progress notes, observation charts, pathology and microbiology results, and prescription sheets. The use of antibiotics was considered as surgical prophylaxis if mentioned in preoperative assessment documents and postoperative if prescribed after surgery. With regards to current antibiotic use, all antibiotics were documented using the ATC classification [7]. Data collection included details of the antibiotic therapy regimen including the start date, dose, route of administration, and the indication for antibiotic therapy. The results of radiological, microbiological, and pathological investigations available at the time of the data collection were also studied to help assess the appropriateness of therapy. The privacy of patients was ensured by decoding all data. No patient was interviewed so no patient consent was needed for the study.

### Appropriateness of antibiotics

Institutional guidelines on antibiotic use had not been developed in any surveyed hospital despite Lahore being the capital city. In addition, there are no national guidelines currently available in Pakistan. Furthermore, there is also no currently established mechanism to implement any guidelines once available within hospitals in Pakistan, such as a Drugs and Therapeutic Committee, antibiotic guideline groups, or antibiotic stewardship groups.

Consequently, antibiotic prescribing guidelines from the British National Formulary were used as the standard to evaluate the appropriateness of prescribing antibiotics [21]. The British National Formulary (BNF) was selected over other possible prescribing guidance references because it is designed for quick reference and is convenient and easy to use [5]. It is also presently available in Pakistan for free via the Pakistan Pharmacist Association. In addition, it is also readily available online, and as paper copies and on mobile applications, and used as a reference in other LMICs [5].

To check antibiotic appropriateness, antibiotic selection, dosage form, dose, frequency, duration, contraindication, hypersensitivity, and antibiotic susceptibility reports were considered against BNF guidance. Antibiotic treatment was considered inappropriate on the basis of the wrong dosage regimen (dosage form, dose, frequency, or duration) or the wrong indication (selection based on indication, spectrum, contraindication, or hypersensitivity) or both.

Data were analyzed using Statistical Program for Social Sciences (SPSS version 20). Simple descriptive statistics were applied to analyze patterns of infection, antibiotic use and the appropriateness of antibiotic prescribing among hospitalized patients. We did not break the figures down into public and private hospitals because we wanted a general picture across both sectors before looking further at specific hospital sites and categories.

### Ethics approval and consent to participate

The study was approved and registered from the Ethics committee on human research, University College of Pharmacy, University of the Punjab, Lahore, Pakistan, under the registration number (HEC/1000/PUCP/1925I). Since the study did not contain any individual person’s data in any form (including any individual details, images or videos), the ethics committee has granted an exemption from consent to be issued before accessing their files. The head of departments of various wards and their staff physicians and nurses were informed about the study and they allowed the principal investigators (ZS, UA) to conduct the study in the respective wards.

### Results

The demographic and clinical properties of the study population are summarized in Table 1. A total of 1185 patients were included with almost an equal number of male (*n* = 595) and female (*n* = 590) patients. A total of 2022 antibiotics were given for 1185 patients. Patients receiving single antibiotic treatment (43.4%) or combination therapy (56.6%) were studied for the appropriateness of their antibiotic treatment. Data collectors gathered the data from a number of wards in the hospitals, including gastroenterology (25.8%), pulmonology (23.2%) and other specialized departments as listed in Table 1.

**Table 1:** Demographic & clinical characteristics (*n* = 1185)

Parameters	Frequency (*N*)	Percentage (%)
*Hospital*		
Public sector	636	53.7
Private sector	549	46.3
*Gender*		
Male	595	50.2
Female	590	49.8
*Type of therapy*		
Single	514	43.4
Combination	671	56.6
*Wards*		
Gastroenterology ward	306	25.8
Pulmonology ward	279	23.5
Nephrology ward	272	22.9
General ward	174	14.8
Orthopedic ward	154	12.9
*Appropriateness of therapy*		
Appropriate	353	29.7
Inappropriate	832	70.3

*n* = Total No. of patients involved in this study

Out of total of 1185 patients on antimicrobial therapy, only 29.7% received appropriate treatment according to the BNF (Table 1). Whereas, approximately two-thirds of the study population (70.3%) had at least one inappropriate antimicrobial prescribed. The highest level of inappropriate antibiotic use (78.5%) was seen in patients with skin and soft tissue infections (Table 2). Patients with respiratory tract infections were considered as having inappropriate therapy on 62.8% of occasions. The lowest level of inappropriate antimicrobial prescribing was seen among patients with bloodstream infections where only 41.2% were seen as inappropriate. Pre-operative prophylaxis was given in 12% of patients with 67.6% having an inappropriate selection.

**Table 2:** Indication and inappropriateness of antibiotic use among patients involved in study (*n* = 1185)

Indication	Patient receiving antibiotics		Inappropriate *N* (%)
	Frequency (*N*)	Percent (%)	
Skin and soft tissue infection	363	30.7	285 (78.5)
Respiratory tract infection	323	27.2	203 (62.8)
Urinary tract infection	183	15.4	138 (75.4)
Gastrointestinal infection	140	11.8	96 (68.6)
Pre-operative Prophylaxis	142	12.0	96 (67.6)
Blood stream infection	34	3.0	14 (41.2)

Table 3 depicts the antimicrobials that were prescribed to hospitalized patients. Cephalosporins were prescribed most frequently (46.1%), out of which 3rd generation cephalosporins, e.g., ceftriaxone, were the most prescribed (78.1%). The second most commonly prescribed antimicrobials were the aminoglycosides (15.6%) followed by the penicillins (14.0%) and fluoroquinolones (9.5%). Then the frequency of antibiotics use was analyzed at ATC level 5, as shown in Table 3. The five most commonly used antibiotics among the hospitalized patients included ceftriaxone (21.0%) followed by amikacin (15.2%), cefoperazone plus sulbactam (11.4%), ciprofloxacin (6.4%) and metronidazole (5.9%). Among the penicillins, amoxicillin plus clavulanic acid was the most prescribed (5.6% of antibiotics) while clarithromycin was the most prescribed macrolide (2.4% of all antibiotics).

**Table 3:** Antibiotics prescribed to hospitalized patients (*n* = 2022)

Antibiotic (ATC codes)	Frequency prescribed (*N*)	Percentage (%)
*Penicillins*	283	14
Narrow and broad spectrum	120	42.4
Ampicillin (J01CA01)	14	0.7
Amoxicillin (J01CA04)	27	1.3
Penicillin v (J01 CE08)	79	3.9
Combination with β-lactamase inhibitor	163	57.6
Amoxicillin and clavulanic acid (J01CR02)	114	5.6
Piperacillin + tazobactam (J01CR05)	49	2.4
*Cephalosporins*	933	46.1
1st generation	114	12.2
Cephalexin (J01DB01)	31	0.2
Cefazolin (J01DB04)	86	4.3
Cephradine (J01DB09)	25	1.3
2nd generation	83	8.9
Cefuroxime (J01 DC02)	72	3.6
Cefaclor (J01 DC04)	11	0.5
3rd generation	729	78.1
Cefotaxime (J01DD01)	23	1.4
Ceftazidime (J01DD02)	20	0.9
Ceftriaxone (J01DD04)	423	21
Cefixime (J01DD08)	32	1.6
Cefoperazone and salbactum (J01DD62)	231	11.4
4th generation	7	0.80
Cefepime (J01DE01)	7	0.3
*Carbapenems*	31	1.5
Imipenem (J01DH51)	9	0.4
Meropenem (J01DH02)	22	1
*Macrolides*	59	2.9
Erythromycin (J01FA01)	4	0.2
Clarithromycin (J01FA09)	48	2.4
Azithromycin (J01FA10)	7	0.3
*Aminoglycosides*	315	15.6
Gentamicin (J01GB03)	8	0.4
Amikacin (J01GB06)	307	15.2
*Fluoroquinolones*	193	9.5
Ciprofloxacin (J01MA02)	130	6.4
Levofloxacin (J01MA12)	17	0.8
Moxifloxacin (J01MA14)	41	2
Ofloxacin (J01MA01)	3	0.1
Norfloxacin (J01MA06)	2	0.09
*Others Antibacterials*	208	10.4
Vancomycin (J01XA01)	80	4.0
Metronidazole (J01XD01)	119	5.9
Linezolid (J01XX08)	9	0.5

Table 4 was built on Tables 1 and 2 by looking further at inappropriate indications or dosage or both of all antibiotics prescribed. Out of the total prescribed, 1283 (63.5%) antibiotic prescriptions had either an inappropriate indication or dosage or both. Cephalosporins were extensively prescribed among patients (Table 3), and in many cases, this was inappropriate (67.2%). Most of the first-generation cephalosporins (80.7%) were inappropriately prescribed mainly because of inappropriate dosages. Penicillins were given to 283 patients, out of which 201 (71.0%) were prescribed either the wrong indication or dosage or both. There was the least inappropriate use of aminoglycosides (35.8%) (Table 4).

**Table 4:** Inappropriate antibiotic Usage (*n* = 2022)

Antibiotics	Inappropriate indication *N* (%)	Inappropriate dosage *N* (%)	Inappropriate treatment *N* (%)
Penicillins	173 (61.1)	140 (49.5)	201 (71.0)
Narrow/broad spectrum	81 (67.5)	92 (76.7)	102 (85.0)
With β-lactamase inhibitor	92 (56.4)	48 (29.5)	99 (60.7)
Cephalosporins	470 (50.4)	418 (44.8)	627 (67.2)
1st generation	37 (32.5)	72 (86.7)	92 (80.7)
2nd generation	32 (38.5)	49 (59.1)	66 (79.5)
3rd generation	395 (54.2)	293 (40.2)	463 (63.5)
4th generation	6 (85.7)	4 (57.1)	6 (85.7)
Carbapenems	20 (76.6)	18 (58.1)	22 (70.9)
Macrolides	37 (62.7)	29 (49.2)	44 (74.6)
Aminoglycosides	67 (21.2)	84 (26.6)	113 (35.8)
Fluoroquinolones	71 (36.8)	108 (55.9)	124 (64.2)
Others Antibacterial	67 (32.2)	133 (63.9)	152 (73.1)

## Discussion

The appropriate use of antibiotics plays an important role in reducing the burden of AMR and establishing a cost-effective healthcare system. Disappointingly, 70.3% of patients in our study received inappropriate antimicrobial therapy, much higher that reported in previous studies conducted in Australia and Turkey [22, 23]. In addition, in Namibia where only 38% of antibiotic prescriptions did not comply with current national guidelines [16] and in Ghana where the appropriateness of the indication for ceftriaxone in a leading hospital was 86% [17]. Surprisingly, inappropriate prescribing of antibiotics for bold stream infections was found to be only 41.2% in this study. This may be due to blood stream infections being seen as more life threatening compared to other types of infection and consequently more attention is paid to them by senior clinicians. This may need further investigation as we cannot say this with certainty.

In our study, we found that third-generation cephalosporins were the most frequently prescribed antimicrobial, and more than half (67.2%) were prescribed inappropriately. However, this is similar to studies in South Africa where poor compliance to indications, administration, and current guidelines was seen for pre-operative prophylaxis in children [24], and in Botswana and Kenya where there was a high rate of inappropriate antimicrobial prescribing post-operatively to help prevent surgical site infections [25, 26]. A study in the US also reported that an incorrect diagnosis was the main reason for inappropriate antimicrobial use in 309 out of 500 patients [27]. Our findings are also similar to other studies published from Pakistan, which showed high inappropriate antibiotic use. In these studies, 88 to 97.6% of antibiotics were prescribed to the patients without a request for a culture test [20, 28, 29]. A cross-sectional study on physician prescribing practices in Pakistan also reported that more than 70% of antibiotics were prescribed by the doctors on patient demand, exacerbated by the fact that the majority of the physicians in the study had not received any kind of training on rational antibiotic prescribing practices [30].

Despite the efforts of the WHO towards developing and implementing strategies to enhance appropriate antimicrobial use, good antimicrobial prescribing practices are still not followed in a number of LMICs [31, 32]. The WHO has also emphasized antimicrobial stewardship programs (ASP) including patient and prescriber education, delayed prescribing, guideline implementation, laboratory testing, and decision support systems, although ASPs can be a particular challenge in LMICs enhanced by manpower issues and available funding [14]. However, this is starting to be addressed [33].

Healthcare providers should also carefully evaluate medication appropriateness and should know about the benefits and risks before prescribing antibiotics to patients [34]. Pertinent knowledge gaps among prescribers need to be addressed with updated prescribing guidelines and continual medical education to minimize the unnecessary use of antibiotics in hospital settings [16, 35]. Research programs, educational sessions by healthcare experts in collaboration with Government officials and professional training can also enhance appropriate prescribing habits among healthcare practitioners alongside the instigation of ASPs among hospitals in Pakistan [36]. Appropriate documentation of medication records among patients receiving antibiotics can also help reduce the over the use of antibiotics by providing a timely reminder of current usage patterns and their rationale and avoiding issues such as missed doses [7, 37]. There should also be a key focus on diagnostic facilities, audits on drug dispensing and prescribing as well as a liaison among academia and healthcare professionals to improve future care based on rational decision making. Education among medical and pharmacy students should also focus on applied pharmacology and clinical pharmacy, including infection and the appropriate use of antibiotics to address key concerns [11, 19]. The presence of pharmacists in hospitals can also favourably influence antimicrobial use in collaboration with other healthcare practitioners through Pharmacy and Therapeutic Committees (PTCs) and helping to develop ASPs in hospitals where these have not existed before [33]. The presence of infectious disease specialists in hospitals may also further help to minimize irrational antibiotic prescribing [38]. Consequently, professional training of healthcare practitioners, execution of essential medicine lists, active PTCs and ASPs, as well encouraging adherence to nationally agreed guidelines can all help to reduce inappropriate antibiotic use in hospitals [14].

We are aware of a number of limitations with this study. Firstly, the information collected was dependent on the knowledge and training of individual data collectors. Whilst each of these data collectors was provided with detailed information, variability between data collectors could not totally be excluded. Secondly, the appropriateness of antibiotic selection was based on the assumption that the diagnosis was accurate as CST reports are not in place in most of the hospitals. This is always a limitation of this type of study in the majority of LMICs. Thirdly, treatment outcomes were not checked as our primary focus was the appropriateness of the antibiotic used. Fourthly, the educational background of the doctors and other medical staff was not considered in this study, neither was disaggregating the findings from private from public hospitals, as we wanted to gain baseline data. Lastly, although this longitudinal survey gave a detailed view of the appropriateness of antibiotic prescribing in the four hospitals surveyed, which were representational of Lahore, we cannot be fully confident that our findings are replicated throughout Pakistan. However, despite these limitations, we believe our findings are robust, providing direction to key stakeholders in Pakistan in the future to improve antibiotic use in hospitals. This will be followed up.

## Conclusion

Inappropriate antimicrobial use was frequently observed among the studied hospitals in Lahore. More than half of the patients had inappropriate antimicrobial therapy, and cephalosporins were frequently prescribed to these patients. This inappropriate use can be minimized through educational interventions for healthcare practitioners, institutional guidelines for appropriate antimicrobial use, implementation of antimicrobial stewardship programs and development of strategies by healthcare professionals and Government officials to combat unnecessary and overuse of antimicrobials in hospitals. These are considerations for the future and will be followed up in future research.

## Data Availability

The datasets used and/or analyzed during the current study are available from the corresponding author on reasonable request.

## Abbreviations

AMR: Antimicrobial resistance
ATC: Anatomical Therapeutic Chemical
CDC: Centers of Disease Control
LMICs: Low and middle countries
ICD: International Classification of Diseases
ENT: Ear Nose Throat
